# Melioidosis in Malaysia: A Review of Case Reports

**DOI:** 10.1371/journal.pntd.0005182

**Published:** 2016-12-22

**Authors:** Paul Vijay Kingsley, Mark Leader, Nandika Suranjith Nagodawithana, Meghan Tipre, Nalini Sathiakumar

**Affiliations:** 1 Pantai Hospital Ipoh, Ipoh, Malaysia; 2 School of Public Health, University of Alabama at Birmingham, Birmingham, Alabama, United States of America; Instituto Butantan, BRAZIL

## Abstract

**Background:**

Melioidosis is a tropical infectious disease associated with significant mortality due to early onset of sepsis.

**Objective:**

We sought to review case reports of melioidosis from Malaysia.

**Methods:**

We conducted a computerized search of literature resources including PubMed, OVID, Scopus, MEDLINE and the COCHRANE database to identify published case reports from 1975 to 2015. We abstracted information on clinical characteristics, exposure history, comorbid conditions, management and outcome.

**Results:**

Overall, 67 cases were reported with 29 (43%) deaths; the median age was 44 years, and a male preponderance (84%) was noted. Forty-one cases (61%) were bacteremic, and fatal septic shock occurred in 13 (19%) within 24–48 hours of admission; nine of the 13 cases were not specifically treated for melioidosis as confirmatory evidence was available only after death. Diabetes mellitus (n = 36, 54%) was the most common risk factor. Twenty-six cases (39%) had a history of exposure to contaminated soil/water or employment in high-risk occupations. Pneumonia (n = 24, 36%) was the most common primary clinical presentation followed by soft tissue abscess (n = 22, 33%). Other types of clinical presentations were less common—genitourinary (n = 5), neurological (n = 5), osteomyelitis/septic arthritis (n = 4) and skin (n = 2); five cases had no evidence of a focus of infection. With regard to internal foci of infection, abscesses of the subcutaneous tissue (n = 14, 21%) was the most common followed by liver (18%); abscesses of the spleen and lung were the third most common (12% each). Seven of 56 males were reported to have prostatic abscesses. Mycotic pseudoaneurysm occurred in five cases. Only one case of parotid abscess was reported in an adult. Of the 67 cases, 13 were children (≤ 18 years of age) with seven deaths; five of the 13 were neonates presenting primarily with bronchopneumonia, four of whom died. Older children had a similar presentation as adults; no case of parotid abscess was reported among children.

**Conclusions:**

The clinical patterns of cases reported from Malaysia are consistent for the most part from previous case reports from South and Southeast Asia with regard to common primary presentations of pneumonia and soft tissue abscesses, and diabetes as a major risk factor. Bacteremic melioidosis carried a poor prognosis and septic shock was strong predictor of mortality. Differences included the occurrence of: primary neurological infection was higher in Malaysia compared to reports outside Malaysia; internal foci of infection such as abscesses of the liver, spleen, prostate, and mycotic pseudoaneurysms were higher than previously reported in the region. No parotid abscess was reported among children. Early recognition of the disease is the cornerstone of management. In clinical situations of community-acquired sepsis and/or pneumonia, where laboratory bacteriological confirmation is not possible, empirical treatment with antimicrobials for *B*. *pseudomallei* is recommended.

## Author Summary

Melioidosis is a bacterial infection occurring in tropical regions of the world. It has been regularly reported from Southeast Asia and Northern Australia and in recent years, reported from South Asia, China and Brazil. The disease can affect any organ in the body, and commonly presents as a lung infection and/or multiple abscesses in internal organs; diabetes is the most common predisposing factor. Melioidosis is associated with a high rate of death because of the early spread of infection to the blood. Although the general clinical presentations of the disease are similar across regions, some regional differences are reported. Therefore, we reviewed case reports of melioidosis from Malaysia. The cases reported from Malaysia were similar to cases reported from South and Southeast Asia with regard to main presentations of pneumonia and soft tissue abscesses and diabetes as a major risk factor; spread of infection to the bloodstream resulted in a greater likelihood of death. Differences were a higher occurrence of neurological presentations, and abscesses of prostate gland, liver and spleen and aneurysms of major blood vessels than previously reported. Abscess of the parotid gland in children was not common. Early diagnosis and treatment including initiating treatment in suspicious cases is crucial to prevent death.

## Introduction

Melioidosis, caused by the gram-negative *Burkholderia pseudomallei* bacillus, is an infectious disease associated with significant mortality due to an early onset of fulminant sepsis. The disease is endemic in Southeast Asia and Northern Australia and is being increasingly reported in other tropical regions of the world such as India, China, Brazil etc [1, 2]. Most information on the epidemiology and risk factors come from studies conducted in Australia and Thailand. Although the general characteristics of the disease are similar, some regional differences in clinical presentations are reported [1–4]. Parotid abscess seen in 30% to 40% of Thai children has been reported only in one case in Australia. On the other hand, prostatic abscess found in about 20% of Australian males has been rarely reported elsewhere [2]. Similarly, brain stem encephalitis with flaccid paralysis noted in 4% of cases in Northern Australia is reported in only 0.2% of Thai population [1]. Thus, there is a need to determine the clinical pattern of the disease in areas of known and potential endemicity to aid physicians in early recognition and prompt treatment. With this in view, we reviewed and synthesized information from all published case reports of melioidosis in Malaysia.

## Materials and Methods

We conducted a computerized search of literature resources including PubMed, OVID, Scopus, MEDLINE and the COCHRANE database to identify all published case reports of melioidosis originating from Malaysia using key search terms, “melioidosis” and “Malaysia.” Overall, we identified 38 papers spanning the time period, 1975 to 2015. We retrieved all papers and excluded four papers for the following reasons: two papers pertaining to animal melioidosis; one paper wherein the case reported was previously included in a case series report; and a paper that reported two cases who acquired melioidosis following the 2004 Sumatra-Andaman tsunami in Sumatra and were treated in Sumatra and subsequently, in Kuala Lumpur. Thus, we reviewed 34 papers [5–38] (individual case reports, n = 25; and case series, n = 9) and abstracted the following information into an EXCEL worksheet: year of publication, case demographic information, history of environmental exposure, preexisting co-morbid conditions, clinical presentation, laboratory diagnosis, treatment and outcome.

The following definitions were used in this review. Acute infection was defined as illness with symptoms for less than two months duration on presentation [39]. Chronic infection was defined as illness with symptoms for more than two months duration on presentation [39]. Relapse was defined as the reappearance of culture-confirmed melioidosis after resolution of symptoms and completion of at least the intensive intravenous phase of antibiotic therapy for the initial melioidosis presentation [39]. A recurrent infection was a new episode of melioidosis occurring after full clinical recovery or convalescence; recurrent infection may be either due to a relapse or to a re-infection with a new strain [40]. Clinical presentations were classified into the following primary diagnostic groups: (1) pneumonia—including associated complications such as pleural effusion, lung abscess; (2) soft tissue infections—infections of non-skeletal tissue surrounding or supporting organs and other structures including subcutaneous tissue, muscle, lymph nodes, blood vessels and soft tissue organs namely the liver or spleen; (3) genitourinary—infection of the urinary and genital systems including the kidneys; (4) osteomyelitis/septic arthritis—infection of bone, joints, ligaments, or cartilage; (5) neurological—brain and spinal cord including meninges and the peripheral nervous system; (6) skin; and (7) no evident focus.

Descriptive analyses were performed. For continuous variables, mean with standard deviation, median and range are presented. Categorical variables are presented as frequency (n) and percentage (%). Where appropriate, differences between proportions were assessed using the chi-square test.

## Results

**Fig 1:**
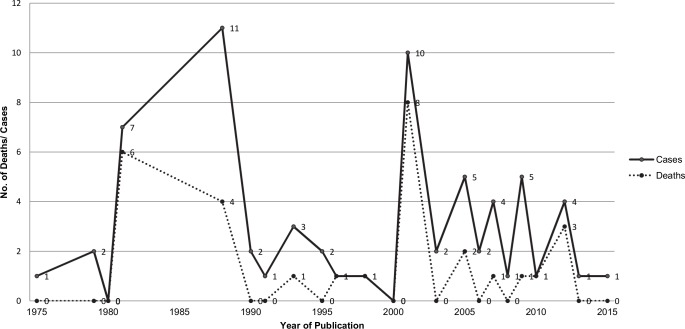
Numbers of melioidosis cases and deaths from published case reports by year of publication, 1975 to 2015.

The earliest paper was published in 1975 and the most recent in 2015. Overall, 67 cases were reported and 29 deaths were attributable to melioidosis (mortality, 43%). An average of two to three cases were reported in about every two years; peaks noted in 1981, 1988 and 2001 are due to case series reports of seven, nine and six cases, respectively (Fig 1). The number of deaths were about half the number of cases and mirror the peaks noted in the cases.

Table 1 presents cases’ demographic characteristics by mortality. Cases’ ages ranged from two days to 73 years, and the median was 44 years. About one-third of cases (n = 21) occurred among persons above 50 years of age and one-fifth of cases (n = 13) were children aged 18 or below. Differences in mortality in these age groups (≤18 and 50+ age groups) compared to the 19–49 year age groups were not significant (53% vs. 33%, p = 0.11). A male preponderance of cases was noted (males, 84%; females, 16%). The mortality among females was two-fold higher than males (73% vs. 38%, p = 0.04); this result was statistically significant. Information on ethnicity, available only for 36 cases, indicated that 13 were Malays, eight were Indians, seven were Chinese, two were Orang Asli, two were migrants, and four cases were of foreign origin (an Australian, a British, a German and a Nepalese). Most cases were reported from Kuala Lumpur (n = 38) followed by Kuantan in Pahang (n = 14) and the remaining from other parts of the country; a slightly higher mortality was noted in Kuantan compared to Kuala Lumpur (57% vs. 45%, p = 0.43). The four cases of foreign nationals were reported outside Malaysia having acquired the disease in Malaysia.

**Table 1:** Distribution of cases by selected characteristics and associated deaths.

	Cases	Deaths
	N (% of total)	N (% in category)
Total	67 (100)	29 (43)
Age (years)		
≤18	13 (19)	7 (54)
19–39	18 (27)	6 (33)
40–49	15 (22)	5 (33)
50+	21 (32)	11 (50)
Mean (SD)	40 (2.4)	40 (4)
Median	44	48
Range (days-years)	2d–73y	2d–69y
Gender		
Males	56 (84)	21 (38)
Female	11 (16)	8 (73)*
Ethnicity		
Malay	13 (19)	4 (31)
Indian	8 (12)	2 (25)
Chinese	7 (10)	5 (71)
Orang Asli	2 (3)	0 (0)
Migrant (Thai, Indonesian)	2 (3)	2 (100)
Foreign	4 (6)	1 (25)
Not reported	31 (46)	15 (48)
Hospital location (city, state)		
Kuala Lumpur†	38 (57)	17 (45)
Kuantan, PahangKuala Lumpur†	14 (21)	8 (57))
Ipoh, Perak	6 (9)	2 (33)
Other‡	5 (7)	1 (20)
Outside Malaysia§	4 (6)	1 (25)

*p<0.05, chi-square test.

†Federal territory located within Selangor

‡Jelebu, Negeri Sembilan; Kota Bharu, Kelantan; Melaka, Malacca; Shah Alam, Selangor and Kajang, Selangor.

§Australia, Germany, Nepal and United Kingdom

Table 2 displays environmental exposures, exposure route and pre-existing comorbid conditions by associated deaths. About 58% of cases had at least one risk factor reported; the mortality among this group was statistically significantly higher than the group without any risk factor reported (64% vs 14%, p = <0.0001). Twenty-six cases (39%) had a high potential for environmental exposure either by engaging in activities that involved contact with soil or water or high-risk occupations. Four cases were involved in search and rescue operations to locate a drowned person in a recreational forest with rivers and waterfalls in Pahang. Three of the four foreign individuals had temporary work assignments in Malaysia and the fourth was visiting Malaysia. High risk occupations included farming or transportation or operating machines such as bulldozers and tractors. Route of exposure was evident in 10 subjects: six subjects had skin wounds sustained while engaging in outdoor activities; and in four cases, the exposure occurred via inhalation following search and rescue operations mentioned above. The latter group of four cases also had leptospirosis; three of the four manifested as pneumonia and had a fatal outcome and one had no evident focus of infection. With regard to pre-existing medical conditions, diabetes mellitus was the most common risk factor (n = 36, 54%) followed by hypertension (15%) and smoking (10%). Difference in mortality among cases with diabetes versus those without diabetes was unremarkable (36% vs. 52%, p = 0.30).

**Table 2:** Cases’ exposure history, risk factors and associated deaths.

	Cases	Deaths
Risk factor	N (% of total)	N (% in category)
Any†	39 (58)	25 (64)
None reported	28 (42)	4 (14)*
Environmental exposure		
High risk activities		
Search and rescue operations	4 (6)	3 (75)
Travel to Malaysia (foreigners)	4 (6)	1 (25)
Residence in a farm	1 (1.5)	1 (100)
High risk occupations		
Trucking/machine operators	9 (13)	3 (33)
Farming/agricultural	8 (12)	2 (25)
Exposure route		
Injury	6 (9)	1 (17)
Inhalation	4 (6)	1 (25)
Preexisting comorbid conditions		
Diabetes	36 (54)	13 (36)
Hypertension	10 (15)	5 (50)
Smoking	7 (10)	4 (57)
Tuberculosis	6 (9)	1 (17)
Chronic heart disease	5 (7)	1 (20)
Chronic renal disease	4 (6)	3 (75)
Autoimmune disorders and/or steroid therapy	4 (6)	2 (50)
Co-infection with leptospirosis	4 (6)	3 (75)
Excessive alcohol use	2 (3)	2 (100)
Acute lymphoblastic leukemia	1 (1.5)	1 (100)
β-thalassemia major	1 (1.5)	0 (0)
Cystic fibrosis	1 (1.5)	0 (0)

* p<0.05, chi-square test.

† A case may have more than one risk factor.

As indicated above, females had a two-fold higher mortality than males. We examined risk factors among the subgroup of women. Of the 11 females, three were children (≤18 years), and the remaining eight ranged from 19 to 61 years of age. Risk factors were reported for nine of the 11 cases and included diabetes (n = 5), systemic lupus erthyematosus (n = 2), juvenile rheumatoid arthritis (n = 1) and residence in a farm (n = 1). Of the 11, eight including the three children had a fatal outcome. Only three adults had a favorable outcome (diabetes, n = 2; systemic lupus erthyematosus, n = 1).

Table 3 provides the distribution of cases by primary diagnostic groups, by bacteremic status and by mortality. Pneumonia (n = 24, 36%) was the most common clinical presentation closely followed by soft tissue abscess (n = 22, 33%); pneumonia had about a three-fold higher mortality than soft tissue abscess (63% vs. 18%, p = 0.003). The other types of clinical presentations were less common: genitourinary (n = 5, 7.5%); neurological (n = 5, 7.5%); osteomyelitis/septic arthritis (n = 4, 6%); and skin (n = 2, 3%). Five of 67 (7.5%) cases had no evidence of a focus of infection. Primary neurological presentations were diverse and included brain abscess (n = 2), subdural empyema (n = 1), meningoencephalaitis (n = 1) or meningitis (n = 1); two of the five had a fatal outcome (brain abscess, n = 1; and sudural empyema, n = 1).

**Table 3:** Numbers of cases and deaths by primary diagnostic groups and by bacteremic status.

Primary diagnostic groups	Total		Bacteremic status					
			Bacteremic		Nonbacteremic		Not reported	
	No.	Deaths	No.	Deaths	No.	Deaths	No.	Deaths
With septic shock	13 **(19%)**	13 (100%)	13	13	-	-	-	-
Pneumonia	10	10	10	10	-	-	-	-
Genitourinary*	1	1	1	1	-	-	-	-
Osteomyelitis/septic arthritis	1	1	1	1	-	-	-	-
Neurological†	1	1	1	1	-	-	-	-
Without septic shock	54 **(81%)**	16 (30%)	28	11	7	0	19	5
Pneumonia	14	5	9	4	2	0	3	1
Soft tissue abscess‡	22	4	8	3	4	0	10	1
Genitourinary*	4	2	3	2	0	0	1	0
Neurological†	4	1	1	0	1	0	2	1
Osteomyelitis/septic arthritis	3	0	3	0	0	0	0	0
Skin infection	2	1	0	0	0	0	2	1
No evident focus	5	3	4	2	0	0	1	1

*Genitourinary with septic shock: prostate abscess (n = 1); without septic shock: prostate abscess (n = 2), perinephric abscess (n = 1) and epididymo-orchitis (n = 1).

†Neurological with septic shock: subdural empyema (n = 1); without septic shock: brain abscess (n = 2), meningoencephalitis (n = 1) and meningitis (n = 1).

‡Soft tissue abscesses included liver abscess (n = 5), mycotic pseudoaneurysm (n = 4), muscle (n = 3), subcutaneous tissue (n = 3), parapharyngeal cellulits (n = 2), pericariditis (n = 1), parotid abscess (n = 1) and lymph node (n = 1).

Of 67 cases, 41 (61%) were bacteremic, seven (10%) were nonbacteremic and bacteremic status was not reported in 19 (28%). Twenty-six of 41 bacteremic cases also had additional supporting laboratory evidence of culture confirmation of *B*. *pseudomallei* from other biospecimens such as pus from abscesses, sputum, bronchial aspirates, joint aspirates or cerebrospinal fluid. Six of the seven nonbacteremic and 11 of the 19 bacteremic status not reported cases had positive cultures on biospecimens other than blood. Most deaths occurred among bacteremic cases compared to nonbacteremic or bacteremic status not reported combined cases (59% vs. 19%, p = 0.002); no death occurred among nonbacteremic cases (0/7).

Septic shock occurred in 13 (19%) bacteremic cases usually within 24 hours of admission; most cases presented with acute respiratory distress syndrome (ARDS). The mortality among cases with septic shock was 100% compared to 30% among cases without septic shock (p = <0.0001).

Besides the primary clinical presentations, it was not uncommon for secondary foci of infection to occur. Overall, 49% of cases had secondary foci of infection. With the exception of skin infection, a secondary focus of infection occurred in all other primary diagnostic groups (Table 4). Secondary soft tissue abscesses were the most common in all primary diagnostic groups followed by secondary pneumonia. With regard to secondary neurological presentations, Parkinsonism was reported in a case presenting with pneumonia, and an acute onset of paraparesis occurred in a case of spinal osteomyelitis with epidural abscess.

**Table 4:** Secondary clinical foci for primary diagnostic groups.

Primary diagnostic group	N	No. with secondary foci (% of total)	Secondary foci presentations (No.)*
Pneumonia	24	14 (58)	Subcutaneous abscess (5), splenic abscess (3), skin infection (3), septic arthritis (2), prostate abscess (1), liver abscess (1), stomach abscess (1), kidney abscess (1), secondary Parkinsonism (1), mediastinal lymphadenopathy (1), mycotic pseudoaneurysm (1)
Soft tissue Abscess	22	8 (36)	Pneumonia (4), prostate abscess (2), splenic abscess (2), subcutaneous abscess (2), liver abscess (1) kidney abscess (1), peritonsillar abscess (1)
Neurological	5	4 (80)	Pneumonia (4)
Genitourinary	5	4 (80)	Pneumonia (2), liver abscess (2), splenic abscess (2), prostate abscess (1), septic arthritis (1), scrotal abscess (1), subcutaneous tissue abscess (1)
Osteomyelitis/septic arthritis	4	2 (50)	Pneumonia (1), liver abscess (1), splenic abscess (1)
Skin infection	2	0 (0)	None
No evident focus	5	1 (20)	Septic arthritis (1), pacemaker infection (1)

*Most cases had more than one secondary focus.

Most cases (n = 63, 94%) were acute in onset and the remaining four cases were chronic in onset. The latter group included two cases of pneumonia, one case of liver abscess and one case of chronic lymphadenopathy; none was a diabetic and all of the four cases were treated successfully. Seven cases (10%) presented as relapses and included primary presentations of mycotic pseudoaneurysm (n = 2), subcutaneous abscess (n = 2), pneumonia (n = 1), liver abscess (n = 1) or no evident focus (n = 1); four of the seven cases were diabetic. All of the cases recovered. Six (9%) cases were recurrences and included clinical presentations as prostatic abscess (n = 2), pericardial effusion (n = 1), psoas abscess (n = 1), lymphadenitis (n = 1) and osteomyelitis (n = 1); five of the six cases were diabetic. One case of prostate abscess developed disseminated disease with septic shock and had a fatal outcome.

Table 5 provides the frequency of internal organ abscesses and other foci of infection. Subcutaneous tissue (21%) was the most common foci of abscesses followed by liver (18%); abcesses of the spleen and lung were the third most common with a frequency of 12% each. Seven of 56 males (13% of males) presented with prostate abscesses either as a primary presentation (n = 3) or as a secondary focus (n = 4) of infection. Five cases presented with mycotic pseudoaneurysms; four presented as a primary presentation and one as a secondary focus. Four cases of brain or spinal cord abscesses were noted. Pericardial effusion occurred in one case and another had infection of the pacemaker device. One case of parotid abscess was noted. Next, details of selected clinical presentations that are known to vary by geographic area are discussed.

**Table 5:** Internal organ abscesses and other soft tissue manifestations.

Site	N (% of total)
Subcutaneous tissue	14 (21)
Liver abscess	12 (18)
Splenic abscess	8 (12)
Lung abscess	8 (12)
Prostate abscess*	7 (13)
Mycotic pseudoaneurysm	5 (7)
Kidney abscess	3 (4)
Muscles–psoas, infrascapular, supraspinatus	3 (4)
Nasopharynx, parapharyngeal, peritonsillar	3 (4)
Lymphadenitis	3 (4)
Brain	2 (3)
Epidural abscess, subdural empyema	2 (3)
Epididymo-orchitis, scrotal abscess*	2 (3)
Parotid abscess	1 (1)
Pericarditis	1 (1)
Stomach	1 (1)
Pacemaker device infection	1 (1)

*Computed for males.

There were seven cases (13%) with prostatic abscesses with ages ranging from 41 to 63 years of age. All of the seven cases had one or more predisposing risk factors: diabetes (n = 6); hypertension (n = 4); tuberculosis (n = 2); or smoking and excessive alcohol use (n = 1). Six of the seven were bacteremic. Presenting symptoms of dysuria, increased frequency of urination or retention of urine were noted only in two cases both of whom were bacteremic and had a fatal outcome (septic shock, n = 1; liver failure, n = 1). In all cases, a diagnosis of prostatic abscess was only made with abdominopelvic computed tomography (CT). None of the cases underwent surgical drainage. All cases of prostatic abscess also had other associated organ abscesses, most often of the liver (n = 5) or spleen (n = 4).

Five cases of mycotic pseudoaneuysms were reported in this review; four of the five were men. The ages ranged from 42 to 66 years of age with a mean of 56 years. The only female case had an underlying systemic lupus erthyematosus and chronic renal failure; two of the men were farmers and another was a foreigner visiting Malaysia. Four of the five cases presented with severe abdominal pain and some evidence of pressure symptoms such as swelling in the lower limbs or paraesthesia. A pulsatile abdominal mass was the predominant clinical sign, and fever was a consistent clinical feature. Diagnosis was made on the basis of CT findings and one case had supplemental angiography; aneurysms were located in the abdominal aorta, external iliac, renal or infrarenal arteries. Four of the five had surgical intervention, and one died post-operatively from anastomotic dehiscence. The fifth patient presented with lung abscess and a thoracoabdominal aortic aneurysm detected on CT. No surgical intervention was performed and the patient died from aneurysmal rupture. All five cases were blood culture-confirmed for *B*. *pseudomallei* with two testing positive from arterial wall culture.

Only one case of parotid abscess was reported in a 64-year old diabetic male who progressed to develop necrotizing fasciitis and pneumonia. Subsequently, he developed septic shock and had a fatal outcome. Pus from the parotid abscess aspirate yielded *B*. *pseudomallei*.

Table 6 presents selected characteristics of 13 paediatric cases, five of whom were neonates. All of the five neonates were bacteremic; four of the five presented with bronchopneumonia and the three of the five developed septic shock. Four of the five neonates died. Older children (n = 8), ranging from 11 to 18 years, presented with pneumonia (n = 3), soft tissue abscess (n = 3), brain abscess (n = 1) or skin ulcer (n = 1). Seven of the eight of the older children had an underlying risk factor; five with underlying immunosuppressive disease and two with potential environmental exposure. Three of the eight children died: one from pneumonia and septic shock; one from cellulitis having underlying acute lymphoblastic leukemia; and one with disseminated melioidosis and septic shock having underlying juvenile rheumatoid arthritis.

**Table 6:** Selected characteristics of pediatric cases (≤ 18 years).

Age (days/years)	Gender (male/female)	Risk factor	Primary presentation/secondary foci	Bacteremic status	Outcome
2 d*†	M		Bronchopneumonia/macular rash	+ve	Died
10 d*	M		Bronchopneumonia	+ve	Died
10 d	M		Meningitis/bronchopneumonia	+ve	Recovered
13 d*	F		Bronchopneumonia	+ve	Died
19 d	M		No evident focus	+ve	Died
11 y	M	Diabetes	Non-healing ulcer	NR‡	Recovered
12 y*†	F	Residence in a farm	Pneumonia	+ve	Died
13 y	M	Acute lymphoblastic leukemia	Cellulitis	+ve	Died
14 y	M		Lymphadenitis with skin abscesses	NR‡	Recovered
15 y	M	β-thalassemia	Abscess in lumbar area	-ve	Recovered
17 y	M	Cystic fibrosis	Pneumonia	-ve	Recovered
18 y†	F	Juvenile rheumatoid arthritis	Pneumonia/splenic and lung abscess	+ve	Died
18 y	M	Farming	Brain abscess	NR‡	Recovered

*With septic shock.

†Culture confirmation obtained after death.

‡NR, not recorded.

Of the overall 67 cases, 14 had no information on treatment. In another 11 patients who were bacteremic (septic shock, n = 9), positive blood culture results for *B*. *pseudomallei* were available only after death; 10 of the 11 patients were treated empirically for community-acquired pneumonia/sepsis with no coverage for *B*. *pseudomallei* infection. Of the 43 remaining patients, most were treated with intravenous ceftazidime during the acute phase and trimethoprim-sulfamethoxazole alone or in combination with chloramphenicol during the eradication therapy.

## Discussion

This review identified 67 cases of melioidosis (mortality, 43%) from individual case reports or as case series reports from Malaysia during the period, 1975 to 2015. About one-third of cases occurred in the 50+ age group. Thirteen cases of children including five neonates were reported. A male preponderance was noted; women had a two-fold higher mortality than men. About two-thirds of cases had at least one risk factor; mortality was significantly higher among those with a risk factor compared to those without a risk factor. Diabetes was the most common risk factor. In two-fifths of cases, a history of exposure either by engaging in activities that involved contact with soil or water or high-risk occupations were elicited. Pneumonia was the most common primary clinical presentation followed by soft tissue abscess; mortality was three-fold higher in cases with pneumonia compared to soft tissue abscess. About 7.5% of cases presented with primary neurologic manifestations with diverse clinical presentations. Bacteremia was reported in three-fifths of cases. Septic shock occurred in one-fifth of cases all of whom were bacteremic with a mortality of 100%; among cases without septic shock, the mortality was 30%. No death occurred among nonbacteremic cases About two-fifths of cases had a secondary foci of infection presenting either as soft tissue abscesses or pneumonia. Internal abscesses were mainly noted in subcutaneous tissue followed by liver, abscesses of the spleen and lung were the third most common. Prostatic abscesses were noted among 13% of males. Mycotic pseudoaneurysms were reported in 7.5% of cases. Only one case of parotid abscess was reported in an adult male.

Table 7 presents salient results of previous papers that describe patterns of the disease in case series from selected areas in Malaysia [41–44], Thailand [1, 45], India [46], and Australia [47]. We synthesized information for Malaysia based on the current review and previously published case series from Malaysia. Overall, cases’ median age was 47 years of age. Males were three times more likely to be affected than females. Diabetes, prevalent in 60% of cases, was the most common risk factor. With regard to primary clinical presentations, pneumonia (44%) was the most common resentation followed by soft tissue abscess/skin (33%). Bone/joint, genitourinary and neurological were less frequent occurring in 9.3%, 6.9% and 6.0% of cases, respectively. Frequencies of internal foci of infection such as abscesses of the liver, spleen, prostate, and mycotic pseudoaneurysms were noted to be higher in this review than previously reported by other Malaysian case series [41–44]. This finding underscores the need for vigilance on the part of physicians to identify and ensure prompt management of such infections. About two-third of cases were bacteremic and septic shock occurred in about 23% of cases. Although some improvements in mortality were noted over time, mortality still remains high. Mortality reported from a case series from 1976 to 1991 was 65%; the average mortality for case series after 2000 was 40%.

**Table 7:** Comparisons of selected results from the present case review and of previously published case reviews of from Malaysia, Australia, Thai land and India.

	Malaysia					Thailand	India	Australia
	Present review (N = 67)	Zueter et al., 2016[41] (N = 158)	Hassan et al., 2010[42] (N = 145)	How et al., 2005[43] (n = 135)	Puthucheary et al., 1992[44] (n = 50)	Suputtamong-kol et al.1999^a^; Cheng & Currie, 2005^b^; (n = 204^a^; 686^b^)*	Vidyalakshmi et al., 2012[46] (n = 95)	Currie et al., 2010[47] (n = 540)
Geographic area	Entire country	Kubang Kerian,Kelantan	Alor Setar, Kedah	Kuantan, Pahang	Kuala Lumpur	Northeastern Thailand^a,b^	Udupi district, Southwestern India	Top End, Australia
Data source	Published Papers	1 hospital laboratory	1 hospital-based registry	2 hospital laborator-ies	1 hospital laboratory	4-hospital case-control study^a^; 6 hospitals^b^	1 hospital laboratory	Prospective Study
Time period	1975–2015	2001–2015	2005–2008	2000–2003	1976–1991	1997^a^; vary between 1978–1985^b^	2005–2010	1989–2009
Inclusion criteria	Acquired disease in Malaysia	Confirmed cases	Confirmed cases	Adults (>18 yrs)	Bacteremia	Culture +ve cases	Culture +ve cases	Culture +ve cases
Demographic								
Age, median (yrs)	44	46*	50	51	44*	58^a^	50	55
Male:female ratio	5.1:1	2.8:1	3.0:1	3.6:1	3.2:1	1.6:1^a^	2.0:1	2.2:1
Risk factor								
Env. exposure %	39	-	-	-	-	85^a^	37	81
Diabetes mellitus %	54	75	57	74	38	60^a^	76	39
No risk factor %	42	16	22	15	24	36^a^	13	20
Primary dx groups								
Pulmonary %	36	41	42	41	58	45^b^	35	51
Soft tissue abscess/skin %	36	28	32	-	34	34	36	16
Bone and joint %	6.0	13	4.8	-	12	5.0^b^	16	4.0
Genitourinary %	7.5	3.2	-	-	10	7.0^b^	3.2	14
Neurologic %	7.5	5.7	4.8	-	6.0	3.0^b^	1.1	2.6
No clinical focus %	7.5	22	-	19	-	-^b^	9.5	11
Primary or secondary								
Liver abscess %	18	12	8.3	3.0	4.0	7.0^b^	7.4	3.0
Splenic abscess %	12	9.5	10	3.0	2.0	2.0^b^	6.3	5.0
Prostate abscess %†	13	2.6	0.9	-	-	0.3^b^	3.2	20
Parotid abscess %	1.5	2.5‡	-	-	-	2.0^b^	1.1	-
Mycotic pseudoaneurysm %	7.5	-	-	-	-	-^b^	-	<1.0
Pericardial effusion %	1.0	-	-	-	2.0%	3.0^b^	2.1	<1.0
Bacteremia %	61	77	52	43	100	58^b^	39	55
Septic shock %	19	34	-	-	16	-^b^	23	21
Mortality %	43	33	34	54	65	38–61^b^	9.5	14

%, calculated as percentage of total number of cases.

-Not recorded.

* Estimated.

†Computed for males.

‡Includes both parotid and lacrimal sac abscesses (n = 4).

Reference a: Suputtamong-kol et al.1999[45]; b: Cheng & Currie, 2005[1].

As noted in the introduction section, variations in clinical presentations of melioidosis have been noted by geographic region. We discuss next, the results of this review in light of findings reported from other Malaysian case series and from case series [41–44] from Thailand [1, 45], India [46], and Australia [47] (Table 7).

In this review, cases’ median age was slightly lower and the male preponderance higher than the findings from other case series. Environmental exposure, reported in 39% of cases, was similar to the case series from India. In contrast, the Thailand and Australian case series report twice as many cases with environmental exposure, 85% and 81%, respectively. In the Australian series, environmental exposure was mostly due to recreational activities and a small proportion due to occupational activities. In the Thai series, 85% of the study group were rice farmers in an endemic area and thus, were considered to have environmental exposure. The proportion of cases with diabetes (54%) is within the range reported from other case series (38% to 75%).

As in other case series, pneumonia was the most common primary clinical presentation involving about one-third of cases; this proportion was similar to the Indian series, but was lower compared to previous Malaysian and other series particularly the Australian series which reports a proportion of 51%. Inhalation as a mode of acquisition of pneumonia has gained importance based on the finding of increased rates of pneumonia following heavy rainfall and extreme weather events [48]; data on weather conditions were not reported in the papers in this review, but acquisition of infection by inhalation was most evident in the four cases involved in search and rescue operations near a waterfall. Primary clinical presentations of soft tissue abscesses combined with skin infection also contributed to one-third of cases. This proportion was consistent with all other series except the Australian series, which reports only 16%. The lack of a unified definition of soft tissue abscess may have deterred accurate comparisons. Soft tissue abscesses and skin infection varied from mild infections to rapidly progressing necrotizing fasciitis. Of note, pneumonia had a three-fold higher morality compared to soft tissue infection. Other primary diagnostic groups were less frequent among all groups with some variations between the groups: bone and joint infection was almost similar to the Thailand and the Australian case series, but lower than the Indian and two previous Malaysian series [41, 44]; genitourinary infection was similar to the Thailand series, but about 50% lower than the Australian series; neurological infection (7.5%) was higher than the Thailand (3.0%), Indian (1.1%), and Australian (2.6%) case series, but consistent with all previous Malaysian series (4.8% to 6.0%). It is important to note that some cases may have no evident focus of infection; this proportion was found to be higher (10% to 22%) when cases are systematically reported from hospital laboratories as in other case series compared to 7.5% in this review of individual cases reports which may be skewed towards publication of severe forms of the disease.

Hepatic and splenic abscesses appear to be more common in Malaysia, Thailand and India as compared to Australia. These visceral abscesses may occur as primary or secondary clinical presentations, and affected organs may not be as tender as in other pyemic infections. Radiological appearance may vary from large abscesses with characteristic “honeycomb” or “Swiss cheese” appearance to a multitude of microabscesses. They rarely require drainage [1].

Reports of neonatal melioidosis in this review emphasize the need to consider melioidosis as a diagnostic possibility in neonates presenting with sepsis, meningitis and/or bronchopneumonia. Information on potential source(s) of transmission was not recorded. One of the five neonates developed symptoms on the day of birth, which suggests that the infection is likely to have been intrauterine. Transplacental infection has been documented in goats; in humans, vertical transmission has been documented only in one case previously [49]. The other four neonates were well at birth and developed symptoms ≥10 days after birth; transmission may have occurred via breast milk or may have been community-acquired. Transmission via culture-positive breast milk has been reported in only one case [50]. Mortality was higher among neonates compared to older children or adults. Older children had clinical presentations similar to that of adults with pneumonia and soft tissue infections occurring more frequently than other primary diagnostic groups. In Thailand, parotid abscess was found to occur among 40% of pediatric cases whereas, it was uncommon in Australia [2]. In this review, and three other Malaysian series [42–44], parotid abscess among pediatric cases was not reported. Zeuter et al [41]. report four cases of parotid gland and lacrimal sac combined in their Malaysian sesries; details on the number of cases with parotid abscess *per se* and their ages were not provided.

Primary neurological infections, occurring in 7.5% of cases, was higher than the proportions reported from Thailand, India and Australia, but was consistent across previous Malaysian series. In this review, the clinical presentations of neurological melioidosis were mainly pyemic such as brain abscess, subdural empyema, epidural abscess etc. Hassan et al [42]. and Puthucheary et al [44]. also report only brain abscesses in their Malaysian series. In Northern Australia, a distinct syndrome of brain stem encephalomyeleitis presenting with flaccid paralysis has been reported in 4% of cases [1]. We found only one reported case of meninogencephalitis in a 21-year old male presenting with hemiparesis. It is to be noted although uncommon, acute onset of neurological symptoms and signs should alert the physician to investigate for melioidosis in endemic and potentially endemic areas.

Prostatic abscess in melioidosis appears to have a differing regional distribution varying from a high of 20% in Northern Australia to a low of 0.3% in Thailand. This review noted prostate abscesses in 13% of males occurring most commonly in association with other intra-abdominal abscesses usually of the liver and spleen. Only two of seven patients were symptomatic and all cases were diagnosed by CT. Of the four previous Malaysian case series, two report a low occurrence of prostate abscess [41, 42] and the other two [43, 44] did not report any case with prostate abscess. It is possible that prostatic abscesses are under-diagnosed as patients are often asymptomatic and CT may not be routinely done. The high occurrence of prostatic abscesses in the Australian series has provided the rationale for routine imaging in that region. The Australian series also found that prostatic abscesses most often required surgical drainage as compared to other internal abscesses that responded well to medical therapy alone. In this review, none of the prostatic abscesses underwent surgical drainage. Treatment protocols may need to consider mandating CT in patients with urinary or abdominal symptoms and to consider surgical drainage of prostatic abscesses.

Mycotic aneurysms are infections of the arterial wall occurring as sequelae of bacteremia. With the advent of antibiotics, mycotic aneurysms are very rare. In a series of 2,585 patients treated for aortic aneurysms, only 22 aneurysms (0.9%) were mycotic [51]. *S*. *aureus* is the most common pathogen associated with mycotic aneurysms followed by *S epidermidis*, non-typhoidal salmonella and streptococcus; *B*. *pseudomallei* as a causal agent for mycotic aneurysms is thus, very rare. Because of the high risk of rupture and death, as in all mycotic aneurysms, mycotic aneurysms due to *B*. *pseudomallei* should be resected regardless of size along with appropriate antibiotic therapy [52]. Early diagnosis is the cornerstone of effective treatment. As symptoms may be frequently nonspecific, a high index of clinical suspicion is the mainstay of early diagnosis [53]. Diagnosis may be established by CT, magnetic resonance imaging, transesophageal echocardiogram or aortography [54]. This review found five cases of mycotic aneurysms due to *B*. *pseudomallei*. Currie et al. [47] report two cases of mycotic aneurysms from Australia. None were reported from Thailand or India. We compared the Malaysian case reports to that of a case series of six cases of *B*. *pseudomallei*-related mycotic aneurysms from Singapore [55]. There was no striking difference between the two series with the exception that fever was not a consistent clinical feature in the Singapore series. The Singapore series reports that all six cases were men, with a mean age of 60 years. Two men had diabetes; and one man had history of soil exposure. Most cases presented with abdominal pain; fever was not a consistent feature. The diagnosis was confirmed by CT, aneurysms were most often located in the infrarenal aorta. *B*. *pseudomallei* was isolated in blood cultures for all cases and from the aortic aneurysmal wall in four cases. All cases underwent surgery and intravenous antibiotic therapy followed by maintenance therapy; one case died from an aortoenteric fistula.

Mortality was much higher in Southeast Asian region (range, 33% to 65%) compared to that reported from India (9.5%) and Northern Australia (14%). The finding of 11 (16%) cases in this review who died before a definitive diagnosis was made and empirically treated with a broad coverage for community-acquired pneumonia/sepsis not including coverage melioidosis, underscores the crucial need for vigilance on part of the physicians to cover for *B*. *pseudomallei* in clinical scenarios of community-acquired pneumonia or sepsis in endemic areas.

Relapses and recurrences of melioidosis may indicate incomplete or inadequate microbial treatment; however, it has been noted relapses and recurrences may occur in immunocompromised patients despite the full course of microbial therapy [56]. Of the 13 cases with relapses/recurrences in this review, 11 had an immunocompromised condition (diabetes, n = 9; systemic lupus erythematosus, n = 1; smoking/chronic alcoholism, n = 1).

Some strengths of this review are noted. This review identified a higher occurrence of primary neurological infection and certain internal foci of infection than previously reported. Further, the finding of confirmatory diagnosis of melioidosis available only after death in some cases, warrants prompt empirical management in suspicious cases. Thus, although case reports have less scientific merit than randomized clinical trials or meta-analyses, they are extremely valuable for the management of emerging or neglected infectious diseases [57]. A general limitation of conducting a review of case reports such as this review is that the information available in each case may not be uniform and may exclude relevant information. Case reports may tend to emphasize unusual findings and have complete work-up for cases which may not be representative of all cases in the region/country. Further, such reviews are limited by the small numbers of cases. Disease registries overcome the limitations of case reports.

In view of the significant mortality, implementing melioidosis registries in all endemic areas appears to be justifiable. Registries can provide health care professionals and researchers with accurate information on the clinico-epidemiologic patterns, and in tracking trends in the incidence, mortality and treatment of melioidosis. Also, using uniform criteria for reporting cases and primary diagnostic groups will increase the specificity of reporting and improve the comparability of information reported from different geographic regions. To our knowledge, Pahang is the only one of 13 states in Malaysia to have a melioidosis registry [58].

In conclusion, the clinical patterns of cases reported from Malaysia are consistent for the most part from previous case reports from South and Southeast Asia with regard to: pneumonia is the most common primary presentation followed by soft tissue abscesses; diabetes is a major risk factor; bacteremic melioidosis carries a poor prognosis; and septic shock is a strong predictor of mortality. Differences included: primary neurological infection was higher in Malaysia compared to reports outside Malaysia; and internal foci of infection such as abscess of the liver, spleen,prostate, and mycotic pseudoaneurysms were higher than previously reported in the region. No parotid abscess was reported among children. Finally, melioidosis is a disease affecting different organ systems with varying clinical patterns. Because of the protean manifestations, the disease is often misdiagnosed and mistreated. Our previous paper discusses pitfalls and optimal approached to diagnosing melioidosis [59]. Early recognition is the cornerstone of management. In clinical situations of clinically highly probable or possible cases particularly in community-acquired sepsis and/or pneumonia, where laboratory bacteriological confirmation is not possible, empirical treatment with antimicrobials for *B*. *pseudomallei* is recommended.

## Supporting Information

S1 Checklist PRISMA Checklist (PDF)

S1 Flowchart PRISMA Flowchart (PDF)
