# Supplementary material for: Melioidosis in Malaysia: A Review of Case Reports
Source: PLoS Negl Trop Dis. 2016 Dec 22;10(12):e0005182. doi: 10.1371/journal.pntd.0005182 (PMC5179056; doi:10.1371/journal.pntd.0005182)
Supplement: S1 Flowchart — (PDF) [file pntd.0005182.s002.pdf]

PRISMA 2009 Flow Diagram

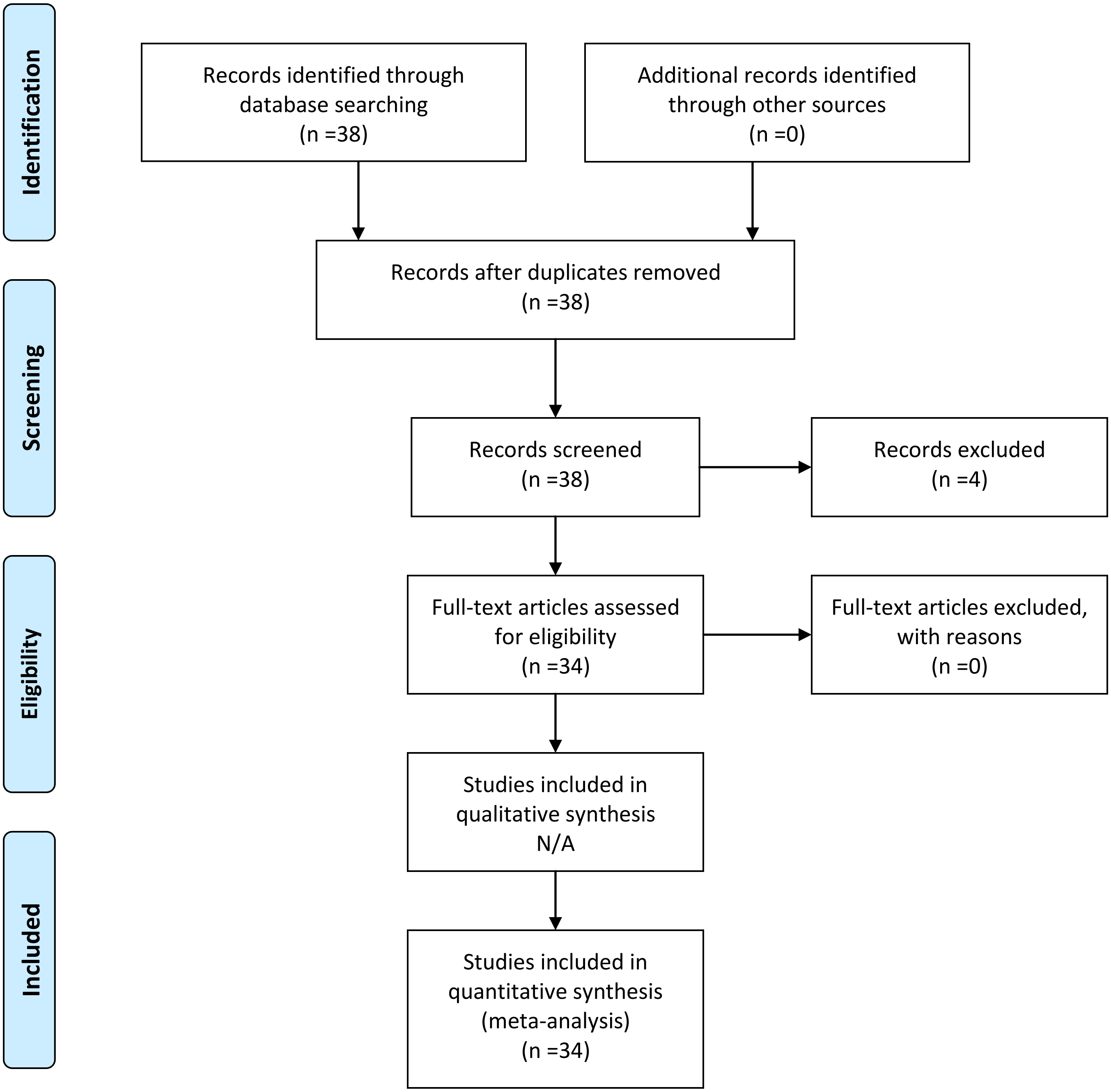

From: Moher D, Liberati A, Tetzlaff J, Altman DG, The PRISMA Group (2009). *P*referred *R*eporting *I*tems for *S*ystematic Reviews and *M*eta-*A*nalyses: The PRISMA Statement. PLoS Med 6(7): e1000097. doi:10.1371/journal.pmed1000097

For more information, visit www.prisma-statement.org.
